# Trends in HIV-1 prevalence and risk behaviours over 15 years in a rural population in Kilimanjaro region of Tanzania

**DOI:** 10.1186/1742-6405-4-23

**Published:** 2007-10-16

**Authors:** Elia J Mmbaga, Akhtar Hussain, Germana H Leyna, Carol Holm-Hansen, Kagoma S Mnyika, Noel E Sam, Elise Klouman, Knut-Inge Klepp

**Affiliations:** 1 Department of Epidemiology and Biostatistics, Muhimbili University of Health Sciences, Dar es Salaam, Tanzania; 2 Department of General Practice and Community Medicine, University of Oslo, Oslo, Norway; 3 Department of Nutrition, Faculty of Medicine, University of Oslo, Oslo, Norway; 4 Norwegian Institute of Public Health, Oslo, Norway; 5 Kilimanjaro Christian Medical Centre, Moshi, Tanzania

## Abstract

**Background:**

Monitoring dynamics in HIV-1 infection and risk behaviours is important in evaluating, adjusting and scaling up prevention programmes. The objective of this study was to estimate trends in the prevalence of HIV-1 infection and risk behaviours over 15 years in a rural village population in Kilimanjaro region of Tanzania using repeated population-based cross-sectional surveys.

**Methods:**

Four rounds of HIV-1 sero-epidemiological and behavioural surveys were completed during 1991 to 2005 in the study village. House-to-house registrations of people aged 15–44 years with an address in the village were conducted before each survey. All consenting individuals were then interviewed for pertinent risk behaviours and tested for HIV-1 seropositivity.

**Results:**

Participation proportions ranged from 73.0% to 79.1%. Overall, age and sex-adjusted HIV-1 prevalence increased from 3.2% in 1991 to 5.6 % in 2005 (relative increase 75.0%; p_trend _< 0.001). The increase was significant for both men and women (p_trends _< 0.001) and more evident among women aged 35–44 years (2.0% to 13.0%, p_trend _< 0.001). Among participants aged 15–24 years a decrease in number of sexual partners was observed with a corresponding stable HIV-1 prevalence. Participants aged 25–44 years continued to report multiple sexual partners, and this was corroborated with increased HIV-1 prevalence trend (4.0% to 9.0%, p_trends _< 0.001). Among men aged 25–44 years and women aged 15–24 years significant increases in condom use were observed (p_trend _< 0.01).

**Conclusion:**

The HIV-1 prevalence seems to have increased among older participants but remained stable among younger participants. Encouraging trends toward safer sex practices were observed among young participants, while only modest behavioural changes were seen among the older participants. Prevention efforts in rural areas need to be intensified and to address people of all ages.

## Background

In recent years, a number of countries with generalised Human immunodeficiency Virus (HIV) epidemic have reported declining prevalence in some population and places and stabilization in others [1-7]. Variation in the spread of HIV infection has increased the demand for data on HIV prevalence and sexual behaviour trends to inform and evaluate HIV prevention activities. Various measures have been employed in attempting to monitor the epidemic in different countries where women attending antenatal clinics (ANC) have been most commonly studied. However, the extent to which trends from antenatal clinics reflect those in the general population have been extensively questioned due to differential HIV testing and urban clinic biases[8,9]. ANC data also lack behavioural information necessary in interpreting the HIV estimates. Despite current efforts to expand surveillance systems in ANC and inclusion of a national representative sample, general population studies are crucial to supplement ANC data [10].

Prevalence estimates are a function of incidence rates, mortality and migration and in mature epidemics the use of incidence rates seems appropriate [11]. However, incidence data are difficult to obtain due to cost and time. In addition, new laboratory tests to detect recently acquired HIV infection are not widely available. This makes prevalence estimates of HIV infection and sexual risk behaviour from representative population samples appropriate measures of the extent and development of the epidemic [10,12]. Therefore, in the context of a rapid scale-up of HIV prevention, care and treatment programmes, it is important to document the magnitude and trends of the HIV epidemic. This is essential for monitoring the dynamics of the epidemic, but also in order to be able to evaluate and, whenever necessary, adjust preventive efforts and document their impact. Moreover, the recommendation by the World Health Organization to implement "second generation" surveillance calls for the monitoring of sexual behaviour in order to assist in explaining the trends in HIV prevalence [12].

The purpose of this study was to investigate the trends in adult HIV-1 prevalence levels from 1991 to 2005 in a rural village population in Tanzania. Furthermore, changes in reported sexual risk behaviours during this period are presented by age groups and sex.

## Methods

### Study area and population

The study was conducted in Oria, a rural village located 30 kilometers south of Moshi town at the foot of Mount Kilimanjaro, Kilimanjaro region, Tanzania. The village is the first stop on the railway from Moshi to Dar es Salaam and located a few kilometers from the Tanzanian-Kenyan border. Oria is a rural village with most of the villagers being peasant farmers, laborers, craftsmen and some engage in seasonal work at the nearby rice plantations. A rice irrigation scheme constructed by the Japanese Government in early 1990's offers opportunities for rice cultivation and business among the villagers. The Chagga, Pare and Kahe are the largest ethnic groups in the village. These tribes are considered indigenous in Kilimanjaro region. Other smaller tribes coming from different part of the country mostly the Sambaa, Zigua, Maasai and Safa are also present. The village has two primary schools and one ordinary secondary school. Two health centers serves the health needs of the villagers and traditional medicine are still popular in this community.

### Survey procedures

The village was first surveyed in 1991 [13] and the second, third and fourth follow-up surveys were conducted in 1993, 1997 and 2005, respectively. Eligibility in all the surveys was based on having a residential address in the village.

In 1991 all individuals aged 15–44 years living in the village were registered by the research team through house-to-house visits and invited to participate in the study. The list was cross-checked with the available village census list to ensure complete enumeration of all eligible individuals. A follow-up study was conducted in 1993. This survey aimed at establishing a cohort hence only those individuals who participated in the 1991 survey were listed and invited to participate. Although attendance was high, data from only 38.4% of individuals was able to be matched[13]. The third and fourth follow-up surveys were conducted in 1997 and 2005, respectively. During the 1997 survey, the age range was limited to 15–36 years. The forth survey conducted in 2005 was similar to the first and second (1991 and 1993) surveys where all individuals aged 15–44 years were included. As in the first enumeration, the house-to-house registration list was cross-checked with the village census list to ensure all individuals of a given age range were registered. Before participation at each survey, all eligible individuals received information regarding the study aims and procedures, and those who agreed to participate provided an informed consent. A structured questionnaire was administered in a private place to ensure confidentiality. Data on socio-demographic and behavioral risk factors for HIV-1 infection were collected. The interviews were then followed by pre-test counseling and sample collection for HIV-1 testing. In 1991, 1993 and 2005 surveys blood samples were used for HIV-1 testing while in 1997 saliva samples were used. Following post-test counseling, test results were issued to respondents in person and those who were HIV-1 positive were offered medical follow-up. At all surveys, individuals who did not participate were followed at home three times before declared as "non-participants". The main reasons for non-participation were fear of partner violence (for women whose husband's were not available) and short-term absence of the respondents from their village address.

All the surveys were approved by the Ethical Committee of the Ministry of Health in Tanzania and the Norwegian Committee for Medical Research Ethics. The village government of Oria granted permission for the study. Individual health education was given to correct any misconception regarding HIV/AIDS and free access to treatment for various medical conditions was provided by the research team during the survey periods. This treatment was provided to all participants and non-participants and their families and was not a condition for participation.

### Testing strategies

Although the HIV-1 testing reagents were different at different times, the same testing strategy (algorithm) was adopted in all surveys. Details of the blood testing strategy has been presented in detail elsewhere [14]. In summary, a standard algorithm that uses two recombinant enzyme-linked immunosorbent assay (ELISA) systems was employed. All discordant or doubtful sera were confirmed by Western blot (Wb; Organon. Epitope, Beaverton, OR, USA). A total of 11.2% of the sera collected in 1991 were subjected to quality control testing at the Centre for International Health, University of Bergen. Excellent agreement was found between the original test results obtained at Kilimanjaro Christian Medical Centre and that from University of Bergen [13,14].

For the saliva HIV-1 screening, the samples were collected by Omni-Sal saliva collection devices (Saliva Diagnostic Systems; Vancouver, WA). The Omni-Sal device consists of an absorbent membrane on a plastic stick specifically designed for collecting oral fluids. The same two ELISA systems were used in HIV-1 antibody detection. In this saliva testing, Bionor HIV 1&2 (Bionor AS; Skien, Norway) and Vironostika HIV-1 Uniform II enzyme linked immunoassay (EIA) (Organon Teknika International, Brussels, Belgium) were used. The Bionor HIV1&2 assays is a rapid EIA based on a novel paramagnetic particle technology developed for use with serum, plasma, whole blood or saliva samples. The Vironostika assay uses the standard EIA format and is designed for use with saliva specimens.

All discordant or doubtful ELISA results were confirmed using Orasure Western Blot (Organon Teknika International, Brussels, Belgium). The OraSure Western blot is a qualitative, more specific test for HIV-1 antibodies in saliva found to be reactive by the Vironostika EIA and other saliva-based assays. The correlation between saliva and serum based tests have been extensively investigated. The sensitivities and specificities of saliva HIV testing have been reported to range from 97.2–100% and 99.7–100% respectively equivalent to those obtained with blood [15-17]. A comparison study conducted in Tanzania found a sensitivity and specificity of 100% between saliva and blood samples[18]. Studies have also revealed that variation in the clinical settings (temperature, lighting and physical layout) in the collection of saliva samples does not affect the performance of saliva samples in relation to blood [19]. This indicates that HIV test results from the two samples could be compared.

### Statistical methods

The response proportion for each survey was calculated by dividing the number of participants by the number of listed eligible in that particular survey. Age and sex-specific prevalence's for the four surveys were estimated using all definitive HIV-1 serological results for each survey. In this estimation, the numerator for the prevalence were the number of adults with an HIV-1 seropositive status and the denominators were number of residents tested (HIV-1 seropositive and seronegative) at that particular survey.

Trends in the HIV-1 prevalence across a number of demographic factors were assessed. Trends in a number of sexual risk behaviours were also examined and these were presented separately for participants aged 15–24 years and those aged 25–44 years. This was due to the observed differences in HIV-1 prevalence trends among these groups. For the calculation of age and sex-adjusted prevalence estimates, district population census figures were used as a standard [20]. To test for significance in trends, the χ^2 ^test for trend in proportion was performed using Epi-Info version 6.03 (Centers for Disease Control and Prevention, Atlanta, Georgia, USA). The student t-test was used to compare means for continues variables and one-way analysis of variance (ANOVA) was used to compare means of the four surveys among participants aged 15–36. In these cases a Statistical Package for Social Sciences (SPSS) for windows version 13.0 (SPSS Inc., Chicago, IL, USA) was used. All reported p-values were 2-tailed and the Confidence Intervals (CI) were at 95% level.

## Results

### Attendance

During the four surveys, totals of 1152, 809, 895 and 1528 individuals participated in 1991, 1993, 1997 and 2005, respectively. The overall response proportions were 76.4%, 76.9%, 79.1% and 73.0%, respectively. The mean age of those attending did not significantly differ from the mean age of all individuals listed in the village (Table 1). For participants, women were significantly older than men in the first three surveys but his was reversed in the fourth survey where men were relatively older than women. In the 1997 survey, only individuals aged 15–36 years were included. ANOVA indicated that the mean age of all participants aged 15–36 years did not differ in all the surveys (p = 0.546 for men and p = 0.397 for women).

**Table 1:** Attendance by sex and survey among adults aged 15–44 years in rural Kilimanjaro region of Tanzania

	1991		1993§		1997*		2005	
				
	M	F	M	F	M	F	M	F
Number listed (N)	708	799	414	635	533	596	852	1241
Mean age (SD)	25.6 (7.5)	26.4 (7.8)	26.6 (8.4)	27.8 (8.3)	23.2 (8.1)	24.5 (8.0)	28.5 (8.6)	28.1 (7.6)
Participants (n)	516	636	318	491	403	492	614	914
Mean age (SD) (all participants)	25.7 (8.3)	26.3 (8.0)	26.5 (8.6)	28.0 (8.6)	22.5 (7.0)	24.1 (7.3)	28.7 (8.8)	27.9 (8.6)
†Mean age (SD) (participants aged 15–36 years)	23.2 (6.0)	23.1 (6.3)	23.4 (6.0)	23.0 (6.1)	23.3 (7.0)	23.2 (7.3)	23.5 (6.6)	23.4 (6.1)
Participation rate (%)	73.0	79.5	76.9	76.9	75.6	82.6	72.1	73.7

N: Listed as having permanent address in the village during the period of the survey.

SD: Standard deviation.

*1997 survey included individuals aged 15–36 years only.

§Only individuals who participated in the 1991 survey and available in the village in 1993 were registered.

† p = 0.546 for men and p = 0.397 for women (From one-way ANOVA).

### Trends in HIV-1 prevalence estimates

In all the surveys, more than 98.0% of the participants accepted HIV-1 testing. Table 2 presents trends in HIV-1 prevalence by sociodemographic characteristics. Overall age and sex-adjusted prevalence of HIV-1 infections increased significantly from 3.2% (95% CI: 3.1–3.3) in 1991 to 5.6 % (95% CI: 5.4–6.1) in 2005 (absolute increase 2.4%, relative increase 75.0%; p_trend _< 0.001). The yearly relative increase in the overall age and sex-adjusted prevalence was 5.0%. Women aged 35–44 years recorded the largest increase (2.0% to 13.0%; p_trend _< 0.001). The increase in prevalence was also evident among individuals aged 25–44 years (4.0% in 1991 to 9.0% in 2005, p_trends _< 0.001). By contrast, HIV-1 prevalence remained stable among young people aged 15–24 years (2.1%, 2.2%, 3.0%, 2.1% for 1991, 1993, 1997 and 2005 respectively, p_trends _= 0.957). Age adjusted HIV-1 prevalence increased significantly for both sexes as displayed in Figure 1 and Table 2. From 1991 to 2005, the prevalence increased from 1.8% to 3.2% (relative increase 77.8%) and from 4.4% to 8.0% (relative increase 81.8%) for males and females, respectively (p_trends _< 0.001 for both sexes). In all surveys, women had a significantly higher prevalence of HIV-1 than men (1.2 to 2.5 times higher in each survey) and the infection peaked at an earlier age in women compared to men (Table 2 and Figure 1). A significant increase in HIV-1 prevalence was seen among individuals who were married or cohabiting. Individuals who reported to have been divorced or separated had a borderline significant increase in prevalence. Moreover, we recorded a significant increase in prevalence among Catholics and Muslims in this community. All village tribes were at increased risk of HIV-1 infections. Individuals who had primary education or lower, as well as those who were farmers showed a significant increase in HIV-1 prevalence (Table 2).

**Table 2:** Trends in the prevalence of HIV-1 infection by sociodemographic characteristics during 1991, 1993, 1997 and 2005 surveys in rural Kilimanjaro, Tanzania

Sociodemographic Characteristics	1991		1993§		1997*		2005		p-value for trend test
					
	Number Prevalence		Number Prevalence		Number Prevalence		Number Prevalence		
	Tested	(%)	Tested	(%)	Tested	(%)	Tested	(%)	
*Age groups (years) Men*									
15–24	262	0.4	158	0.0	254	2.1	223	0.0	0.829
25–34	126	3.1	101	3.0	106	2.9	201	3.7	0.268
35–44	129	2.7	59	2.7	41	4.0	190	5.9	0.153
All ages for men	516	1.9	318	1.9	403	3.0	614	3.2	< 0.001
*Age groups (years) Women*									
15–24	261	3.7	253	4.0	253	2.7	352	3.5	0.839
25–34	193	7.5	162	9.2	172	6.5	333	7.5	0.167
35–44	181	2.0	76	2.4	69	6.7	229	13.0	< 0.001
All ages- women	636	4.4	491	5.2	492	5.3	914	8.0	< 0.001
*Marital status*									
Single	447	2.7	302	4.5	381	2.7	508	1.9	0.147
Married/cohabiting	575	3.3	405	3.7	415	4.5	875	7.3	< 0.001
Divorced/separated	130	8.0	102	9.0	99	17.1	145	15.9	0.058
*Religion*									
Catholic	322	2.3	224	1.8	272	4.1	404	7.0	< 0.001
Protestant	279	5.2	223	5.1	213	4.4	471	6.9	0.228
Muslim	551	3.0	362	3.9	410	3.9	653	5.8	0.015
*Tribe*									
Chagga	321	3.3	173	4.1	242	2.5	438	7.6	0.005
Pare/Kahe	459	3.5	272	2.4	432	4.9	563	5.4	0.041
Other	372	3.1	364	3.6	221	4.5	527	6.6	0.039
*Education level*									
No education	93	1.2	55	4.3	31	3.2	161	11.2	0.003
Primary education	985	3.2	692	3.7	812	4.7	1250	5.8	0.001
Secondary +	74	5.3	62	1.7	52	1.9	117	5.7	0.278
*Occupation*									
Farmer	1037	3.2	704	3.8	792	4.0	1327	6.3	< 0.001
Employed	68	3.4	61	3.4	53	8.8	49	2.1	0.767
Business	47	6.3	44	4.5	50	6.0	152	10.8	0.180

**Total prevalence#**	**1152**	**3.2**	**809**	**3.6**	**895**	**4.1**	**1528**	**5.6**	**< 0.001**

* 1997 survey included individuals aged 15–36 years only.

§ Only individuals who participated in the 1991 survey and available in the village in 1993 were registered.

# Age and sex- adjusted HIV-1 prevalence

**Figure 1:**
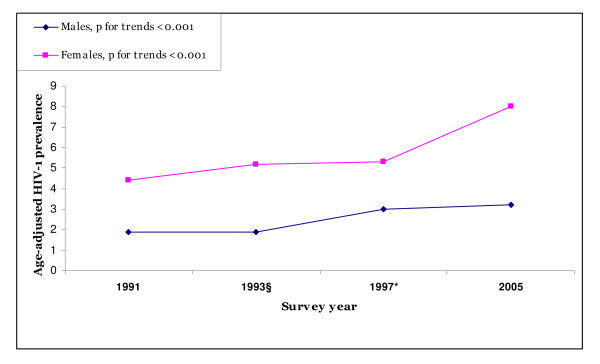
Trends in HIV-1 prevalence by sex and survey among adults aged 15–44 years in rural Kilimanjaro region of Tanzania. The figure illustrates the increased HIV-1 trends by sex from 1991 to 2005. The increased prevalence trends were significant for both sexes (p_trends _< 0.001 for both sexes). * 1997 survey included individuals aged 15–36 years only. § Only individuals who participated in the 1991 survey and available in the village in 1993 were registered.

### Trends in sexual risk behaviours

Trends in reported sexual risk behaviours among individuals aged 15–24 and 25–44 years are presented in Tables 3 and 4, respectively. The proportion of participants aged 15–24 reporting being sexually active (ever had sex) ranged from 66.2% to 87.1%. Across surveys, virtually all (98% to100%) of those aged 25–44 years reported being sexually active. From 1991 to 2005, decreased trends in the proportions of young men reporting being sexually active was observed (Table 3). No equivalent decrease was observed among young women or older age group. The compiled reported median age at first sex (age at sexual debut) was lower among young men compared to young women (15.0 for men versus 17.5 for women, p < 0.001). Conversely, the proportion of young men aged 15–24 who reported to ever have had sex before the age of 15 years decreased significantly during the study period (relative decrease 37.6%, p_trend _< 0.001). No decreased trend in the corresponding proportions was observed among young women. Women were more likely to report polygamous marriage than men in this community with an increased trend among young women (p_trends _< 0.001) (Table 3).

**Table 3:** Trends in risk behaviors by sex and survey among adults aged 15–24 years in rural Kilimanjaro region of Tanzania

Variables	Category	1991	1993	1997	2005	p-value for trend test
						
		N = 523	N = 411	N = 511	N = 575	
Sexually active (%)						
	Men	87.1	75.3	72.7	69.7	< 0.001
	Women	69.3	78.7	66.2	73.5	0.650
Age at first sex-median in years ^a^						
	Men	15.0	16.0	15.0	15.0	0.768
	Women	18.0	18.0	17.0	17.0	0.564
Participants aged 15–24 years who had sex at age ≤ 15 years (%)						
	Men	61.4	54.6	48.2	38.3	< 0.001
	Women	28.1	27.3	32.4	22.0	0.104
Age at first marriage/cohabiting-median in years ^b^						
	Men	22.0	21.0	20.5	21.0	0.868
	Women	19.0	18.0	18.0	18.0	0.798
Polygamous marriage (%)						
	Men	0.0	0.6	0.8	0.1	0.961
	Women	3.6	3.0	6.2	11.3	< 0.001
No. partners past 4 weeks						
	Men: ≥ 2 partners	18.1	4.2	6.0	5.0	< 0.001
	Women: ≥ 2 partners	5.2	3.8	4.9	2.7	0.024
No. of partners past year ^c^						
	Men: ≥ 2 partners	59.0	48.2	32.5	30.4	< 0.001
	Women: ≥ 2 partners	15.0	13.8	12.4	6.4	0.003
No. of partners past 5 years ^c^						
	Men: ≥ 2 Partners	73.5	71.1	47.7	51.0	< 0.001
	Women: ≥ 2 Partners	35.2	33.8	27.0	25.5	0.016
Ever/lifetime used condom (%)						
	Men	39.8	37.8	34.0	42.2	0.800
	Women	21.4	29.6	23.1	37.9	< 0.001
Presently have GDS (%)						
	Men	2.7	2.5	4.8	1.3	0.350
	Women	1.9	2.5	1.6	2.9	0.374
Presently have GUD (%)						
	Men	1.5	1.9	1.2	0.5	0.274
	Women	0.4	0.6	1.2	1.4	0.112
Circumcision	Men	86.6	86.5	-	88.2	0.748

^a ^Median age at first sex from those who ever had sex.

^b ^For those who have ever married/cohabited.

^c^Among those who ever had sex.

GDS: Genital discharge syndrome, GUD: Genital ulcer disease

**Table 4:** Trends in risk behaviors by sex and survey among adults aged 25–44 years in rural Kilimanjaro region of Tanzania

Variables	Category	1991	1993	1997*	2005	p-value for trend test
						
		N = 629	N = 398	N = 384	N = 953	
Sexually active (%)						
	Men	99.6	99.4	98.7	98.5	0.920
	Women	100.0	100.0	99.1	98.4	0.710
Age at first sex-median in years ^a^						
	Men	18.0	18.0	17.0	18.0	0.987
	Women	18.0	18.0	18.0	18.0	1.000
Age at first marriage/cohabiting-median in years ^b^						
	Men	26.0	27.0	24.0	25.0	0.163
	Women	19.0	19.0	19.0	20.0	0.215
Polygamous marriage (%)						
	Men	8.3	12.6	11.5	7.7	0.679
	Women	19.6	11.6	19.7	17.6	0.741
No. Partners past 4 weeks						
	Men	13.8	6.1	14.4	11.2	0.514
	Women	2.2	3.5	2.0	3.3	0.610
No. of partners past year ^c^						
	Men: ≥ 2 partners	38.3	36.4	27.0	35.2	0.522
	Women: ≥ 2 partners	10.6	13.9	13.5	10.1	0.514
No. of partners past 5 years ^c^						
	Men: ≥ 2 Partners	78.0	69.0	45.4	44.2	< 0.001
	Women: ≥ 2 Partners	26.1	29.3	25.8	20.1	0.014
Ever/lifetime used condom (%)						
	Men	32.7	48.3	39.0	50.3	0.002
	Women	26.7	21.9	20.5	28.4	0.069
Presently have GDS (%)						
	Men	2.5	6.3	2.7	3.3	0.392
	Women	2.3	4.9	3.9	4.4	0.132
Presently have GUD (%)						
	Men	1.3	0.6	0.0	2.1	0.227
	Women	1.1	1.1	1.3	1.1	0.349
Circumcision	Men	89.5	90.1	-	92.4	0.283

* 1997 survey included individuals aged 15–36 years only.

^a ^Median age at first sex from those who ever had sex.

^b ^For those who have ever married/cohabited.

^c^Among those who ever had sex.

GDS: Genital discharge syndrome, GUD: Genital ulcer disease

A significant proportion of young men aged 15–24 years reported a reduction in the number of sexual partners in this population (past 4 weeks, relative reduction 87.3%, p_trends _< 0.001, past 1 year; relative reduction 48.5%, p_trends _< 0.001 and past 5 years; relative reduction 30.6%, p trends < 0.001). Similar trends were also observed among young women with corresponding relative reductions of 48.1% (p_trends _0.024), 57.3% (p_trends _0.003) and 27.3% (p_trends _0.016), respectively (Table 3). Apart from a reported reduction in the number of sexual partners during the past 5 years, there were no corresponding trends among participants aged 25–44 years (Table 4).

A significantly increased trend in reported condom use was observed in this population. Almost half of the men and a third of the women in both age groups reported to have ever used a condom at the time of the last survey in 2005. The increased trends were significant among young women (relative increase 77.1%, p_trends _< 0.001) and older men (relative increase 53.8%, p_trends _= 0.002). The prevalence of men circumcision was high in both age groups and remained so during the survey period (Tables 3 and 4). In all surveys, no association was found between circumcision and HIV infection in this population.

## Discussion

The results of this study demonstrated significant increase (75%) in HIV-1 prevalence in this rural population from 1991 to 2005. The increase was mainly due to a statistically significant increase in HIV infection among individuals aged 25–44 years. The increase was particularly prominent in women aged 34–44 years recording the largest increase. Significant trends were observed for both men and women. During the study period the HIV-1 infection prevalence stabilised among those aged 15–24 years, and this finding was supported by an observed trend towards safer sex during the same time period. Except for the increased proportion of those reporting ever having used condom, no corresponding trends towards safer sex were observed for the age group 25–44 years. Increased HIV-1 prevalence trends were also recorded among married participants, all village tribes, those with poor education and those engaging in farming.

Previous studies involving rural populations from within and outside Tanzania have reported increased or higher HIV-1 prevalence[6,7,21-24]. Tanzania Demographic Health Survey (DHS) and recent studies have shown an increased susceptibility and vulnerability to HIV infection among rural communities signalling a potential for a further escalating epidemic [24,25]. Prevalent state of sexually transmitted infections [13] including Herpes Simplex Virus type-2 (prevalence 33.2% in this village, unpublished) render rural population vulnerable for more intensified epidemics. This indicates that while HIV-1 infection might be decreasing in urban areas as reported from various recent studies, it could be taking a different direction in rural areas[1-3,5,7,22,24,26].

The incidence of HIV-1 infection in this population in 1993 was 13.0/1000 person years at risk (PYAR) among women as compared to 4.3/1000 PYAR among men [13]. In the 1991 survey, a high prevalence of HIV-1 infection (8.7%) was recorded among women aged 25–29 years [14]. These individuals are expected to be in the age group 35–44 years in the last survey in 2005. The current observed high HIV-1 prevalence (13.0%) among this group could partly be attributed to an aging cohort effect. However, increased mortality is expected among the older age group thus reducing the HIV-1 prevalence. This would have been the case in our rural population where antiretroviral treatment is not widely available. Therefore, the observed increase in HIV-1 prevalence among women aged 34–44 years and/or participants aged 25–44 years in the background of expected higher mortality could not be solely attributed to an accumulated effect, but partly as an indication of increased HIV-1 incidence among the older age group. This findings are corroborated by reports from Botswana and South Africa which revealed an increased HIV-1 prevalence among individuals aged above 35 years of age [27]. This may indicate the need to improve prevention programmes that address all age groups.

Contrary to our findings, studies in Rakai Uganda and Kagera, Tanzania have reported a decreased HIV prevalence and incidence[2,3]. However, these studies have been conducted in areas considered to be the epicentre of the HIV/AIDS epidemic in Africa where several prevention programmes are operating and the epidemic is more mature. This might explain the observed difference in HIV-1 prevalence between our study area and others [2,3,6,8,22] that involved rural population far from the epicentres.

Reduction in HIV prevalence was observed among educated individuals in this rural population. This has also been reported in other studies in Africa where the role of education both general and HIV/AIDS related in promoting behavioural change has been reported [4,28-30]. Increased HIV prevalence among married individuals has also been described in Tanzania [23]. As the epidemic matures, the rate of discordant couples increases and HIV transmission within long-term relationships is believed to be the core driver of the epidemic [31].

Trends towards safer sex were observed among young people in our population. HIV intervention programmes were provided to youth both in-and-out of school in the study village[30]. The intervention aimed at increasing knowledge and building HIV prevention skills among young people in this community. The observed positive behavioural change might have contributed to curbing the HIV-1 epidemic among young people [32]. In contrast to the encouraging trends among young people, modest behavioural change was observed among the older age group. Substantial proportion of older age group continued to practice risk behaviours probably above the threshold needed to arrest the epidemic as indicated by the increased HIV-1 prevalence among them [31,33]. Therefore, the observed positive trend among young people failed to translate into a reduced overall HIV-1 risk in this population as the risk was simply delayed because behaviour was not changed among older group. An encouraging finding in this village is the observed high rate of male circumcision. This is because recent findings from randomised controlled trials in Kenya, Uganda and South Africa have shown that, men circumcision reduces the risk of HIV acquisition through penile-vaginal sex by 51–60% [34,35]. This could partly explain the disproportionably low HIV prevalence among men as compared to women in this population.

It is noteworthy that comparison in HIV prevalence's from serial cross-sectional surveys is affected by changes in population structure in each survey. A substantial bias in the HIV estimates could arise from different recruitment and population structure especially in the second and third surveys (1993 and 1997). Biased listing and hence participation may have effect on the overall survey estimates. For example, exclusion of older individuals aged 37–44 years in the 1997 survey may have underestimated the true HIV estimates as this age group recorded high prevalence in the 2005 survey. Therefore, the HIV estimates of these two surveys (1993 and 1997) should be interpreted with caution.

We have only been able to report the incidence of HIV-1 infection in the early years of follow-up (1991–1993) in this population [13]. Due to the long duration of the two last follow-up after 1993, it was difficult to match participants between the surveys. Therefore these surveys were considered as independent cross-sectional surveys. The long intervals between follow-up surveys after 1993 made it difficult to match individual data for incidence calculations. However, this long interval had an advantage in the linkage of behavioural data with biological data. It gave time for the effect of any behavioural change to have an effect on the slowly evolving HIV prevalence[31].

Except for the use of saliva samples during the 1997 survey, the same testing strategies (algorithm) were adopted in all the survey rounds. Saliva testing has been found to yield results comparable to serum testing as explained in details in the methodology [15,17-19]. However, we acknowledge the possibility of an underestimation of HIV prevalence estimates when saliva was used. Changes in HIV tests may also influence trends. HIV antibody tests have improved over the years. New generation assays are more accurate and the rate of false-positive results that may ultimately skew prevalence estimates are low. The overall results of improved tests would have yielded low prevalence. Hence, the increased prevalence observed in our study is thus not a testing artefact. Limitations related to the validity of self-reported behavioural data have been reported [7,10,22,36]. Reporting bias due to desirability is more likely to have affected assessment of trend data. The same questions were used in all surveys allowing comparison across surveys. The four surveys produced similar results although desirability biases might have masked a true increase in risk behaviours. Prevalence data may have been affected by migration as migrants are less likely to participate. The differential exclusion of this high risk group may results in underestimation of HIV prevalence [37]. In these surveys, seasonal workers especially high-risk men may have been missed. Therefore, the possible biases to our findings that may arise from missed migrants could not be ruled out in this investigation and warrants further investigation.

## Conclusion

This study provides evidence of increased prevalence of HIV-1 infection in a rural population in Tanzania far from the known epicentre of HIV-1 in Africa. Married or divorced individuals had increased prevalence of HIV-1. Secondary school education or higher education seems to be protective for HIV-1 infection as shown in this and other recent studies. Trends toward safer sex among young people with a positive impact on the HIV-1 epidemic were observed. HIV/AIDS education campaign among youth in the village may have played a role. Older people continued to practice risk behaviours and this was reflected in the increased HIV-1 infection prevalence in this age group. Promoting behavioural change and reducing the practice of multiple sexual partners should be a major focus of HIV-1 prevention efforts in rural areas. For prevention programmes to be effective they should address individuals of all ages.

## Competing interests

The author(s) declare that they have no competing interests.

## Authors' contributions

EJM conceived the study, coordinated data collection and analysis, interpreted the results and drafted the manuscript; AH designed the study, interpreted the data and reviewed the manuscript; GHL designed the study, collected the data, analyzed and interpreted the data and reviewed the manuscript; CHH, KSM, NES and EK participated in data collection, laboratory testing, interpreted the data and reviewed the manuscript, KIK coordinated the design and protocol, analyzed and interpreted the data and reviewed the manuscript. All authors read and approved the final draft of the paper.
